# Plastic Operation upon the Ear

**Published:** 1858-01

**Authors:** Joseph Pancoast

**Affiliations:** Professor of Anatomy in Jefferson Medical College, Philadelphia


ART. IX.—*Plastic Operation upon the Ear.* By JOSEPH PANCOAST, M.D., Professor of Anatomy in Jefferson Medical College, Philadelphia. Reported by WM. M. BREED, M.D.

IN August last, a young man disfigured by the loss of a considerable portion of his right ear, from a bite in a fight, applied to Professor Pancoast to have the deformity relieved by a plastic operation. Two months had elapsed since the occurrence of the injury. The condition of the parts was as follows:—The cicatrix an irregular one, but imperfectly healed, and evidently not in a healthy condition: the amount of loss sustained was fully one-third of the external ear, and this was considerable, as the ears were unusually large and pendulous—that upon the opposite side measuring nearly three inches in length. The entire lobule was gone. A line carried across the inferior portion of the concha to a point on the helix opposite the auditory meatus will afford a good idea of the course of the cicatrix.

The operation was performed in the following manner:—The entire edge of the wound was freshened by paring, and the cartilage removed to a greater depth than the integument; a portion of the cutaneous tissue was then raised from over the upper extremity of the sterno-cleido-mastoid muscle of more than twice the size of the lost part, allowing thus for shrinking, and of such a shape that, when doubled upon itself, it should correspond exactly to the same portion of the sound ear, the pedicle representing the attachment of the lobule. The flap being doubled so as to present both surfaces covered with integument was then secured to the raw edges of the ear by a few interrupted sutures, and well sustained with strips of isinglass-plaster. The parts united kindly except a small point of the posterior edge of the flap, where there was a little sloughing, owing, most likely, to the presence of coagulated blood between its folds. The operation was in every respect a successful one. The attachment of the new lobule being made to take the same position and direction as the natural one rendered the shape of the two ears almost exactly similar.

